# The GLP-1 Receptor Agonist Liraglutide Decreases Primary Bile Acids and Serotonin in the Colon Independently of Feeding in Mice

**DOI:** 10.3390/ijms25147784

**Published:** 2024-07-16

**Authors:** Katsunori Nonogaki, Takao Kaji

**Affiliations:** Division of Diabetes and Nutrition, RARiS, Tohoku University, 6-6-11 Aramakiaza-Aoba, Aoba-ku, Sendai 980-8579, Miyagi, Japan; takao.kaji.d4@tohoku.ac.jp

**Keywords:** GLP-1 receptor agonist, liraglutide, serotonin, tryptophan, bile acids

## Abstract

Liraglutide, a glucagon-like peptide 1 analog used to treat type 2 diabetes and obesity, is a potential new treatment modality for bile acid (BA) diarrhea. Here, we show that administration of liraglutide significantly decreased total BAs, especially the primary BAs, including cholic acid, chenodeoxycholic acid, taurocholic acid, taurochenodeoxycholic acid, glycocholic acid, and β-muricholic acid, in the liver and feces. In addition, liraglutide significantly decreased tryptophan metabolites, including L-tryptophan, serotonin, 5-hydroxy indole-3-acetic acid, L-kynurenine, and xanthurenic acid, in the colon, whereas it significantly increased indole-3-propionic acid. Moreover, the administration of liraglutide remarkably decreased the expression of apical sodium-dependent bile acid transporter, which mediates BA uptake across the apical brush border member in the ileum, ileal BA binding protein, and fibroblast growth factor 15 in association with decreased expression of the BA-activated nuclear receptor farnesoid X receptor and the heteromeric organic solute transporter Ostα/β, which induces BA excretion, in the ileum. Liraglutide acutely decreased body weight and blood glucose levels in association with decreases in plasma insulin and serotonin levels in food-deprived mice. These findings suggest the potential of liraglutide as a novel inhibitor of primary BAs and serotonin in the colon.

## 1. Introduction

Liraglutide, a glucagon-like peptide 1 (GLP-1) analog used to treat type 2 diabetes, increases the postprandial insulin level in a glucose-dependent manner and reduces glucagon secretion, leading to improved postprandial glucose metabolism. In addition, liraglutide delays gastric emptying and induces weight loss through reductions in appetite and energy intake [1,2,3]. Moreover, findings from a recent clinical trial suggest the potential of liraglutide as a new treatment modality for bile acid (BA) diarrhea [4]. The acute effects of liraglutide on blood glucose levels in a fasted condition and the mechanisms by which liraglutide reduces the frequency of stool in BA diarrhea, however, remain unclear. 

Serotonin (5-hydroxytryptamine, 5-HT) is a monoamine derived from tryptophan (Trp). In the periphery, circulating 5-HT is primarily produced in enterochromaffin (EC) cells of the gastrointestinal tract through microbial metabolites, including short-chain fatty acids and secondary BAs [5]. 5-HT is required to maintain normal intestinal peristalsis, and the increased 5-HT in the colon is critically involved in the pathogenesis of inflammatory bowel diseases [6], irritable bowel syndrome [7], and post-cholecystectomy diarrhea [8]. Moreover, 5-HT is involved in glucose metabolism and energy homeostasis [5,9]. We therefore hypothesized that liraglutide may alter Trp metabolites and decrease 5-HT in the colon, which may be involved in the improvement of BA diarrhea and glucose-reducing effects of liraglutide in the fasted state. 

We have previously reported that the administration of liraglutide suppresses food intake and body weight in mice [10,11], and that the anorexic effect of liraglutide occurs within 1 h [10] and sustains for 24 h after the drug administration [11]. To exclude the anorexic effect of liraglutide, we examined the effects of liraglutide on BAs in the liver and feces, tryptophan metabolites in the colon, blood glucose, plasma insulin, 5-HT, and fibroblast growth factor 21 (FGF21), and gene expression involved in BA transport in the ileum of mice deprived of food after intraperitoneal administration of the drug.

## 2. Results

### 2.1. Effects of Liraglutide on Body Weight, Blood Glucose, Plasma Insulin, Serotonin, and FGF21 Levels in Mice Deprived of Food after Intraperitoneal Administration of the Drug

**Figure 1:**
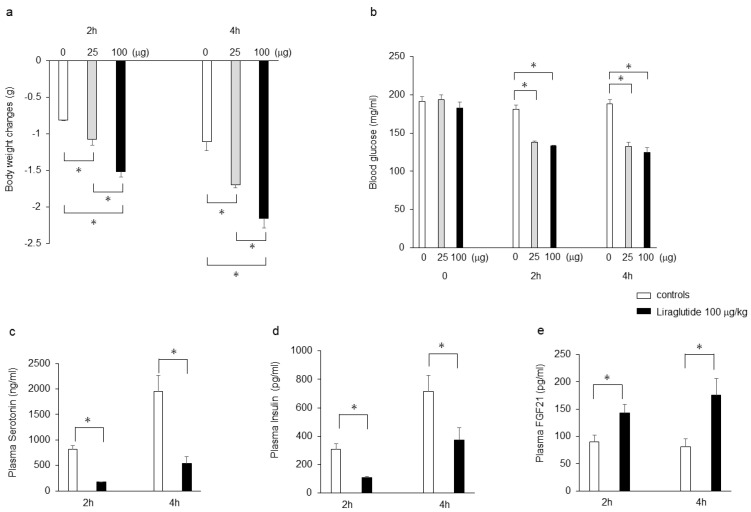
Effects of intraperitoneal injection of liraglutide (25 and 100 μg/kg) or saline on body weight (**a**) and blood glucose levels (**b**) in C57BL6N mice deprived of food after the injection. Effects of intraperitoneal injection of liraglutide (100 μg/kg) or saline on plasma 5-HT (**c**), insulin (**d**), and FGF21 (**e**) levels in C57BL6N mice deprived of food for 2 and 4 h after the injection. Data are presented as the mean ± SEM (n = 6/group). * *p* < 0.05.

Intraperitoneal injection of liraglutide (25 and 100 μg/kg) significantly decreased body weight in a dose-dependent manner compared with saline controls in mice that were food-deprived for 2 h and 4 h after the injection (Figure 1a). In addition, intraperitoneal injection of liraglutide (25 and 100 μg/kg) significantly decreased blood glucose levels, but there were no significant differences between the doses of 25 μg/kg and 100 μg/kg liraglutide (Figure 1b). Moreover, intraperitoneal injection of liraglutide (100 μg/kg) significantly decreased plasma 5-HT (Figure 1c) and insulin (Figure 1d) levels, whereas it significantly increased plasma FGF21 levels (Figure 1e) compared with the saline controls in mice that were food-deprived for 2 h and 4 h after the injection.

### 2.2. Effects of Liraglutide on Tryptophan Metabolites in the Colon of Mice

The administration of liraglutide at 100 μg/kg significantly decreased L-tryptophan (Trp), 5-HT, 5-hydroxy indole-3-acetic acid (5-HIAA), L-kynurenine (KYN), and xanthurenic acid (XA) in the colon of mice that were food-deprived for 2 h after the injection, whereas it significantly increased indole-3-propionic acid (IPA; Table 1).

### 2.3. Effects of Liraglutide on Bile Acids in the Feces and Liver of Mice

The administration of 100 μg/kg liraglutide significantly decreased total bile acids (TBA) and the primary BAs including cholic acid (CA), chenodeoxycholic acid (CDCA), taurocholic acid (TCA), taurochenodeoxycholic acid (TCDCA), glycocholic acid (GCA), and β-muricholic acid (β-MCA) in the colon of mice that were food-deprived for 2 h after the injection, whereas it did not decrease the secondary BAs, including deoxycholic acid (DCA), ursodeoxycholic acid (UDCA), taurodeoxycholic acid (TDCA), and lithocholic acid (LCA) in the feces (Table 2).

The administration of 100 μg/kg liraglutide significantly decreased TBA and the primary BAs including CA, TCA, and β-MCA in the liver of mice that were food-deprived for 2 h after the injection, whereas it did not decrease DCA, TDCA, and LCA in the liver (Table 3).

### 2.4. Effects of Liraglutide on Expression of Genes Involved in BA Transport in the Ileum

**Figure 2:**
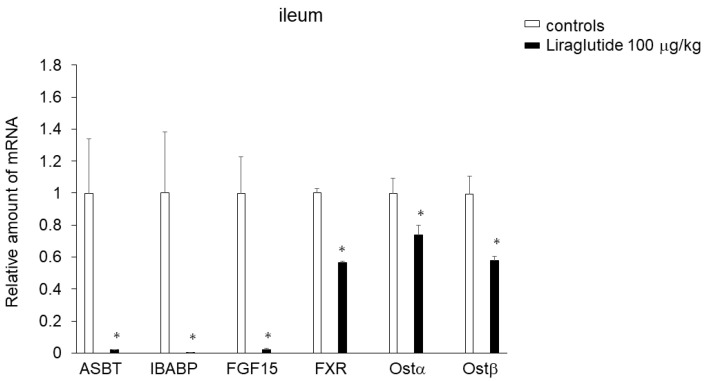
Effects of intraperitoneal injection of liraglutide (100 μg/kg) or saline on expression of genes involved in BA transport in the ileum of C57BL6N mice deprived of food for 2 h after the injection. Data are presented as the mean ± SEM (n = 6/group). * *p* < 0.05.

The administration of liraglutide (100 μg/kg) dramatically decreased the expression of apical sodium-dependent bile acid transporter (Asbt; Slc10a2), which mediates BA uptake across the apical brush border member in the ileum, ileal BA binding protein (IBABP), and FGF15 in the ileum compared with saline controls in mice that were food-deprived for 2 h after the injection. Moreover, liraglutide significantly decreased expression of the BA-activated nuclear receptor farnesoid X receptor (FXR) or the heteromeric organic solute transporter Ostα/β, which induces BA excretion, in the ileum (Figure 2).

## 3. Discussion

The results of the present study revealed that liraglutide acutely decreased total BAs, especially the primary BAs, and 5-HT in the colon of mice deprived of food after the drug administration. The BA-sequestrant colesevelam reduces stool frequency in BA diarrhea [12]. A recent clinical trial comparing the effects of liraglutide and colesevelam on BA diarrhea demonstrated the superiority of liraglutide in reducing stool frequency [4]. BA diarrhea is characterized by excess delivery of BAs to the colon [13]. Although BA diarrhea partially overlaps irritable bowel syndrome with diarrhea, the unconjugated and primary BAs in serum and feces are higher in BA diarrhea [13]. Our findings indicate that the liraglutide-induced decreases in the primary Bas and 5-HT in the colon may contribute to the mechanisms by which liraglutide reduces stool frequency in BA diarrhea.

GLP-1 released from enteroendocrine cells in the small intestine in response to nutrient metabolites reportedly stimulates 5-HT secretion via GLP-1 receptors in enterochromaffin cells of the small intestine and colon [14]. Our results, however, demonstrated that liraglutide decreased Trp, 5-HT, and 5-HIAA in the colon, leading to decreases in plasma 5-HT levels in mice deprived of food after administration of the drug. The liraglutide-induced decreases in total BAs, especially the primary BAs, might contribute to the suppression of gut-derived 5-HT secretion. 

BAs are actively absorbed by the ASBT in distal ileal mucosa. Genetic ablation of ABST, which induces BA uptake in the enterocytes, decreases FGF15 expression in the ileum [15,16,17] and total BAs in the liver [18] of mice. Our findings revealed that liraglutide acutely inhibited the expression of ASBT, IBABP, and FGF15 in the ileum. These findings suggest that the liraglutide-induced suppression of total BAs, especially in primary BAs, in the liver may be due to the inhibition of ASBT expression in the ileum and pathways other than the gut-derived FGF15-mediated hepatic BAs synthesis.

In the present study, despite decreases in the plasma insulin levels, liraglutide acutely decreased blood glucose levels in the food-deprived mice, suggesting that the acute decreases in blood glucose levels induced by liraglutide are independent of feeding and insulin secretion. Despite the increased insulin levels at 4 h, blood glucose levels were unchanged in control mice, suggesting an insulin-resistant state. Liraglutide might therefore reduce insulin resistance. Liraglutide also acutely decreased body weight in the food-deprived mice, suggesting that liraglutide increases energy expenditure. 

The liraglutide-induced decreases in the total BAs, especially the primary BAs, may contribute to the decreases in blood glucose levels and body weight in mice deprived of food after the drug administration. BA sequestrants, including colesevelam, have beneficial effects on glycemic control in subjects with type 2 diabetes and diabetic rodents [19]. The BA-sequestrant colesevelam also suppresses body weight gain in microbiome-humanized mice fed a chow diet or Western diet [20]. 

The inhibition of intestinal ASBT induced by liraglutide may also contribute to the decrease in blood glucose levels and body weight in mice deprived of food after drug administration. Genetic or pharmacologic inhibition of ASBT decreases blood glucose levels associated with decreased insulin resistance and increased hepatic FGF21 expression in mice [16]. The genetic or pharmacologic inhibition of ASBT also suppresses a high-fat diet-induced body weight gain in mice and hamsters [16,17,18]. The increases in plasma FGF21 levels induced by liraglutide may contribute to the decreases in blood glucose levels and body weight. FGF21 reportedly increases glucose uptake in adipose tissue and suppresses hepatic glucose production in mice [21]. FGF21 also increases energy expenditure, fat utilization, and thermogenesis within brown adipose tissue in mice [21].

In addition, the decreases in 5-HT induced by liraglutide may contribute to the decrease in blood glucose levels and body weight in mice deprived of food after the drug administration. Genetic and/or pharmacologic inhibition of tryptophan hydroxylase 1 (TPH1), which induces the decreases in serum and colonic mucosal 5-HT, can improve glucose tolerance without affecting insulin sensitivity [9]. Genetic and/or pharmacologic inhibition of TPH1 also prevents high-fat-diet-induced body weight gain by promoting brown adipose tissue thermogenesis in mice [22]. Increased peripheral 5-HT synthesis induced by nutrients such as by overeating, a high-fat diet, and a high carbohydrate diet, or altered microbiota composition has been suggested as the pathophysiologic mechanisms of obesity-related diseases including metabolic syndrome, non-alcoholic fatty liver disease, type 2 diabetes, and cardiovascular disease [5]. 

Moreover, the increase in IPA induced by liraglutide may contribute to the decrease in blood glucose levels and body weight in mice deprived of food after the drug administration. IPA is a microbial product from tryptophan absorbed from the gut into the bloodstream [23,24]. Type 2 diabetes and obesity likely decrease IPA levels in the serum and/or colonic mucosa [24,25,26], and IPA supplementation exerts beneficial effects on body weight and glucose metabolism [27]. The increase in IPA induced by liraglutide may therefore have beneficial effects on body weight and glucose metabolism. Liraglutide may affect the intestinal microflora, which produces IPA, leading to beneficial effects on metabolic disorders.

The sustainability of these changes induced by liraglutide over an extended period including under meal-stimulated conditions and in diarrhea models, and the effects of liraglutide on intestinal microflora, remain unclear. Further studies will need to determine them in the future.

These findings suggest that the liraglutide-induced decrease in primary BAs and alterations of Trp metabolites in the colon may be involved in the improvement of BA diarrhea and/or the body weight and glucose-reducing effects of liraglutide independently of feeding.

## 4. Materials and Methods 

### 4.1. General Procedures 

Six-week-old male C57BL6N mice were individually housed in cages with free access to water and chow pellets in a light- and temperature-controlled environment (12 h on/12 h off, lights on at 08:00; 20–22 °C) for 1 week before the experiment. 

In the first experiment, 7-week-old mice were intraperitoneally injected with saline or liraglutide (25 and/or 100 μg/kg) and then deprived of food after the treatment. At 2 h and 4 h later, body weights and blood glucose levels were measured.

In the second experiment, 7-week-old mice were intraperitoneally injected with saline or liraglutide (100 μg/kg) and then deprived of food after the treatment. At 2 h and 4 h later, the animals were decapitated, and blood was obtained for the measurement of plasma insulin, 5-HT, FGF21, and total BA levels. The liver, ileum, and colon were dissected to determine mRNA levels, BAs, and tryptophan metabolites at 2 h later. 

The doses of liraglutide used were described previously [21,22]. The experiment was performed between 12:00 and 17:00. 

The animal studies were conducted in accordance with the institutional guidelines for animal experiments at Tohoku University Graduate School of Medicine and all experimental protocols were approved by the institutional ethics committee at Tohoku University.

### 4.2. Blood Chemistry

Whole blood was mixed with EDTA-2Na (2 mg/mL) and aprotinin (500 kIU/mL) to determine the plasma levels of FGF21, 5-HT, and insulin. Plasma insulin, 5-HT, and FGF21 levels were measured by enzyme-linked immunosorbent assay (mouse Insulin ELISA Kit [TMB], AKRIN-011T, Shibayagi, Gunma, Japan, mouse 5-HT; BA E-5900, Labor Diagnostika Nord, Nordhorn, Germany, and mouse FGF21 ELISA kit) as described previously [23,24]. Blood glucose levels were measured using glucose strips (blood glucose monitoring system; Accu-Check, Roche Diagnostics, Tokyo, Japan). 

### 4.3. BA Analysis

BA analysis was performed using a facility service at LSI Medience Corporation, a contract clinical trial company (Tokyo, Japan) as described previously [24]. Briefly, approximately 100 mg of liver and colon tissue was transferred to disruptor tubes supplied by Yasui Kikai (Osaka, Japan) and shaken with iron cones cooled in liquid nitrogen. The tissue powders were suspended with 1 mL of water and 4 mL of methanol. After mixing using a shaker for 15 min, the samples were centrifuged at 1000× *g* for 15 min at room temperature. The supernatants were analyzed by liquid chromatography–tandem mass spectrometry (Nexera X2 LC0AD, 8050, Shimadzu, Kyoto, Japan) equipped with reverse phase LC column (InfinityLab Poroshell 120 EC-C18 2.7 mm, 2.1 mm × 150 mm, Agilent Technologies, Santa Clara, CA, USA). The data were analyzed by LabSolutions (Shimadzu, Kyoto, Japan). The peak areas were normalized by internal standards, and each bile acid concentration was obtained using a standard curve.

### 4.4. Tryptophan and Its Metabolites Analysis

Tryptophan and its metabolites were subjected to analysis by LSI Medience Corporation, a contracted laboratory based in Tokyo, Japan. Briefly, approximately 100 mg of colon tissues was introduced into sample disruptor tubes provided by Yasui Kikai (Osaka, Japan). Subsequently, these tubes were agitated with iron cones that had been pre-cooled in liquid nitrogen. The resulting sample powders were suspended in 500 µL of methanol and vigorously shaken for 15 min, after which centrifugation was carried out at 20,000× *g* for 3 min. The supernatants, constituting 40 µL, were meticulously transferred to 2 mL microtubes. Internal standards were then introduced and combined with the supernatants, followed by the addition of 1000 µL of a 2% formic acid solution to induce protein precipitation. Afterward, we purified the analytes from the supernatant using solid-phase extraction (OASIS MCX, Waters, Milford, MA, USA) and analyzed them using liquid chromatography–tandem mass spectrometry (Ultivo, Agilent, Santa Clara, CA, USA) with a reverse-phase LC column (ACQUITY UPLC HSS T3, 1.8 µm, 2.1 mm × 50 mm, Waters, Milford, MA, USA). The data were processed with Mass Hunter software https://www.agilent.com.cn/en/promotions/masshunter-mass-spec (accessed on 14 July 2024) (Agilent, Santa Clara, CA, USA). We normalized the peak areas using internal standards and determined the concentration of each analyte using a standard curve.

### 4.5. Real-Time Quantitative Reverse Transcription–Polymerase Chain Reaction (RT–PCR)

Total RNA was isolated from mouse liver using the RNeasy Midi kit (Qiagen, Hilden, Germany) according to the manufacturer’s instructions. cDNA synthesis was performed using a Super Script III First-Strand Synthesis System for RT-PCR Kit (Invitrogen, Rockville, MD, USA) with 1 μg total RNA. cDNA synthesized from total RNA was evaluated in a real-time PCR quantitative system (LightCycler Nano Instrument Roche Diagnostics, Mannheim, Germany). The relative amount of mRNA was calculated using β-actin mRNA as the invariant control. Data are shown as fold-change of the mean value of the control group as described previously [28,29]. The primers of RT-PCR are listed in Table 4.

### 4.6. Statistical Methods

Data are presented as mean ± SEM (n = 6). Comparisons between the two groups were performed using Student’s t-test. Comparisons between more than two groups were performed using analysis of variance with Bonferroni’s correction for multiple comparisons. A *p* value of less than 0.05 was considered statistically significant.

## Figures and Tables

**Table 1:** Liraglutide-induced alterations of Trp metabolites in the colon of mice.

(ng/g)	Control	Liraglutide	*p*-Value
Trp	15,620 ± 4608.09	3027 ± 220.4	*p* < 0.05
5-HT	1367 ± 120.7	385 ± 13.66	*p* < 0.05
5-HIAA	462 ± 66.0	266 ± 5.42	*p* < 0.05
KYN	311 ± 56.93	46 ± 0.82	*p* < 0.05
XA	417 ± 100.68	72.1 ± 7.72	*p* < 0.05
IPA	29.9 ± 1.91	652.5 ± 119.94	*p* < 0.05

Trp; L-tryptophan, 5-HT; serotonin, 5-HIAA; 5-hydroxy indole-3-acetic acid, KYN; L-kynurenine, XA; xanthurenic acid, and IPA; indole-3-propionic acid.

**Table 2:** Liraglutide-induced alterations of BAs in the feces of mice.

(nmol/g)	Control	Liraglutide	*p*-Value
CA	291.18 ± 68.59	16.53 ± 1.79	*p* < 0.05
TCA	70.65 ± 23.95	5.42 ± 2.3	*p* < 0.05
TCDCA	2.55 ± 0.75	0.39 ± 0.12	*p* < 0.05
GCA	0.57 ± 0.11	0.07 ± 0.01	*p* < 0.05
αMCA	37.13 ± 11.25	21.48 ± 4.21	NS
βMCA	176.93 ± 62.51	70.60 ± 17.04	*p* < 0.05
DCA	382.08 ± 108.4	407.5 ± 72.23	NS
UDCA	28.16 ± 9.10	31.6 ± 8.73	NS
TDCA	4.54 ± 1.50	1.3795 ± 0.41	*p* < 0.05
LCA	11.88 ± 4.37	20.89 ± 3.89	NS
GDCA	0.27 ± 0.06	0.37 ± 0.10	NS
TBA	1008.78 ± 155.75	439.81 ± 87.28	*p* < 0.05

CA, cholic acid; CDCA, chenodeoxycholic acid; TCA, taurocholic acid; TCDCA, taurochenodeoxycholic acid; GCA, glycocholic acid; αMCA, α-muricholic acid; βMCA, β-muricholic acid; DCA, deoxycholic acid; UDCA, ursodeoxycholic acid; TDCA, taurodeoxycholic acid; LCA, lithocholic acid; GDCA, glycodeoxycholic acid; and TBA, total bile acids; NS, No significant.

**Table 3:** Liraglutide-induced alterations of BAs in the liver of mice.

(nmol/g)	Control	Liraglutide	*p*-Value
CA	2.72 ± 0.14	1.45 ± 0.41	*p* < 0.05
TCA	131.57 ± 23.57	39.55 ± 0.47	*p* < 0.05
TCDCA	2.36 ± 0.15	2.01 ± 0.46	NS
GCA	0.18 ± 0.03	0.16 ± 0.05	NS
CDCA	0.31 ± 0.02	0.24 ± 0.05	NS
αMCA	0.91 ± 0.12	0.78 ± 0.18	NS
βMCA	5.24 ± 0.26	3.57 ± 0.19	*p* < 0.05
DCA	0.21 ± 0.004	0.20 ± 0.01	NS
UDCA	0.25 ± 0.02	0.18 ± 0.08	*p* < 0.05
TDCA	10.28 ± 0.99	8.57 ± 1.82	NS
LCA	0.06 ± 0.02	0.08 ± 0.06	NS
TBA	154.1 ± 23.46	43.54 ± 6.57	*p* < 0.05

NS, No significant.

**Table 4:** The primers of RT-PCR were listed.

GENES	Sense	Antisense
FGF15	ACGGGCTGATTCGCTACTC	TGTAGCCTAAACAGTCCATTTCCT
FXR	CCCCTGCTTGATGTGCTAC	CGTGGTGATGGTTGAATGTC
ASBT	TGGGTTTCTTCCTGGCTAGACT	TGTTCTGCATTCCAGTTTCCAA
IBABP	CAGGAGACGTGATTGAAAGGG	GCCCCCAGAGTAAGACTGGG
Ostα	TACAAGAACACCCTTTGCCC	CGAGGAATCCAGAGACCAAA
Ostβ	GTATTTTCGTGCAGAAGATGCG	TTTCTGTTTGCCAGGATGCTC
β-actin	TTGTAACCAACTGGGACGATATGG	GATCTTGATCTTCATGGTGCTAGG

## Data Availability

Some or all datasets generated during and/or analyzed during the current study are not publicly available but are available from the corresponding author on reasonable request.

## References

[1] Campbell J.E., Drucker D.J. (2013). Pharmacology, physiology, and mechanisms of incretin hormone action. Cell Metab..

[2] Nauck M.A., Quast D.R., Wefers J., Meier J.J. (2021). GLP-1 receptor agonists in the treatment of type 2 diabetes—state-of-the-art. Mol. Metab..

[3] Popoviciu M.S., Păduraru L., Yahya G., Metwally K., Cavalu S. (2023). Emerging Role of GLP-1 Agonists in Obesity: A Comprehensive Review of Randomised Controlled Trials. Int. J. Mol. Sci..

[4] Kårhus M.L., Brønden A., Forman J.L., Haaber A., Knudsen E., Langholz E., Dragsted L.O., Hansen S.H., Krakauer M., Vilsbøll T. (2022). Safety and efficacy of liraglutide versus colesevelam for the treatment of bile acid diarrhoea: A randomised, double-blind, active-comparator, non-inferiority clinical trial. Lancet Gastroenterol. Hepatol..

[5] Nonogaki K. (2022). The Regulatory Role of the Central and Peripheral Serotonin Network on Feeding Signals in Metabolic Diseases. Int. J. Mol. Sci..

[6] Koopman N., Katsavelis D., Hove A.S.T., Brul S., Jonge W.J., Seppen J. (2021). The Multifaceted Role of Serotonin in Intestinal Homeostasis. Int. J. Mol. Sci..

[7] Chojnacki J., Konrad P., Mędrek-Socha M., Kaczka A., Błońska A., Zajdel R., Chojnacki C., Gasiorowska A. (2024). The Variability of Tryptophan Metabolism in Patients with Mixed Type of Irritable Bowel Syndrome. Int. J. Mol. Sci..

[8] Xu Y., Wang J., Wu X., Jing H., Zhang S., Hu Z., Rao L., Chang Q., Wang L., Zhang Z. (2023). Gut microbiota alteration after cholecystectomy contributes to post-cholecystectomy diarrhea via bile acids stimulating colonic serotonin. Gut Microbes.

[9] Martin A.M., Yabut J.M., Choo J.M., Page A.J., Sun E.W., Jessup C.F., Wesselingh S.L., Khan W.I., Rogers G.B., Steinberg G.R. (2019). The gut microbiome regulates host glucose homeostasis via peripheral serotonin. Proc. Natl. Acad. Sci. USA.

[10] Nonogaki K., Kaji T. (2016). The acute anorexic effect of liraglutide, a GLP-1 receptor agonist, does not require functional leptin receptor, serotonin, and hypothalamic POMC and CART activities in mice. Diabetes Res. Clin. Pract..

[11] Nonogaki K., Kaji T. (2018). Liraglutide, a GLP-1 Receptor Agonist, Which Decreases Hypothalamic 5-HT2A Receptor Expression, Reduces Appetite and Body Weight Independently of Serotonin Synthesis in Mice. J. Diabetes Res..

[12] Borup C., Vinter-Jensen L., Jørgensen S.P.G., Wildt S., Graff J., Gregersen T., Zaremba A., Borup Andersen T., Nøjgaard C., Timm H.B. (2023). Efficacy and safety of colesevelam for the treatment of bile acid diarrhoea: A double-blind, randomised, placebo-controlled, phase 4 clinical trial. Lancet Gastroenterol. Hepatol..

[13] Sagar N.M., Duboc H., Kay G.L., Alam M.T., Wicaksono A.N., Covington J.A., Quince C., Kokkorou M., Svolos V., Palmieri L.J. (2020). The pathophysiology of bile acid diarrhoea: Differences in the colonic microbiome, metabolome and bile acids. Sci. Rep..

[14] Lund M.L., Egerod K.L., Engelstoft M.S., Dmytriyeva O., Theodorsson E., Patel B.A., Schwartz T.W. (2018). Enterochromaffin 5-HT cells—A major target for GLP-1 and gut microbial metabolites. Mol. Metab..

[15] Rao A., Haywood J., Craddock A.L., Belinsky M.G., Kruh G.D., Dawson P.A. (2008). The organic solute transporter alpha-beta, Ostalpha-Ostbeta, is essential for intestinal bile acid transport and homeostasis. Proc. Natl. Acad. Sci. USA.

[16] Lundåsen T., Andersson E.-M., Snaith M., Lindmark H., Lundberg J., Östlund-Lindqvist A.-M. (2012). Inhibition of intestinal bile acid transporter Slc10a2 improves triglyceride metabolism and normalizes elevated plasma glucose levels in mice. PLoS ONE.

[17] Ge M.-X., Niu W.-X., Ren J.-F., Cai S.-Y., Yu D.-K., Liu H.-T., Zhang N., Zhang Y.X., Wang Y.C., Shao R.G. (2019). A novel ASBT inhibitor, IMB17-15, repressed nonalcoholic fatty liver disease development in high-fat diet-fed Syrian golden hamsters. Acta Pharmacol. Sin..

[18] Rao A., Kosters A., Mells J.E., Zhang W., Setchell K.D., Amanso A.M., Wynn G.M., Xu T., Keller B.T., Yin H. (2016). Inhibition of ileal bile acid uptake protects against nonalcoholic fatty liver disease in high-fat diet-fed mice. Sci. Transl. Med..

[19] Hansen M., Sonne D.P., Mikkelsen K.H., Gluud L.L., Vilsbøll T., Knop F.K. (2017). Bile acid sequestrants for glycemia control in patients with type 2 diabetes: A systematic review with meta-analysis of randomized controlled trials. J. Diabetes Complicat..

[20] Hartmann P., Duan Y., Miyamoto Y., Demir M., Lang S., Hasa E., Stern P., Yamashita D., Conrad M., Eckmann L. (2022). Colesevelam ameliorates non-alcoholic steatohepatitis and obesity in mice. Hepatol. Int..

[21] Iglesias P., Selgas R., Romero S., Díez J.J. (2012). Biological role, clinical significance, and therapeutic possibilities of the recently discovered metabolic hormone fibroblastic growth factor 21. Eur. J. Endocrinol..

[22] Crane J.D., Palanivel R., Mottillo E.P., Bujak A.L., Wang H., Ford R.J., Collins A., Blümer R.M., Fullerton M.D., Yabut J.M. (2015). Inhibiting peripheral serotonin synthesis reduces obesity and metabolic dysfunction by promoting brown adipose tissue thermogenesis. Nat. Med..

[23] Wikoff W.R., Anfora A.T., Liu J., Schultz P.G., Lesley S.A., Peters E.C., Siuzdak G. (2009). Metabolomics analysis reveals large effects of gut microflora on mammalian blood metabolites. Proc. Natl. Acad. Sci. USA.

[24] Zhang B., Jiang M., Zhao J., Song Y., Du W., Shi J. (2022). The Mechanism Underlying the Influence of Indole-3-Propionic Acid: A Relevance to Metabolic Disorders. Front. Endocrinol..

[25] Tuomainen M., Lindström J., Lehtonen M., Auriola S., Pihlajamäki J., Peltonen M., Tuomilehto J., Uusitupa M., de Mello V.D., Hanhineva K. (2018). Associations of serum indolepropionic acid, a gut microbiota metabolite, with type 2 diabetes and low-grade inflammation in high-risk individuals. Nutr. Diabetes.

[26] de Mello V.D., Paananen J., Lindström J., Lankinen M.A., Shi L., Kuusisto J., Pihlajamäki J., Auriola S., Lehtonen M., Rolandsson O. (2017). Indolepropionic acid and novel lipid metabolites are associated with a lower risk of type 2 diabetes in the Finnish Diabetes Prevention Study. Sci. Rep..

[27] Chen L., Yang Y., Sun S., Xie Y., Pan C., Li M., Li C., Liu Y., Xu Z., Liu W. (2022). Indolepropionic acid reduces obesity-induced metabolic dysfunction through colonic barrier restoration mediated via tuft cell-derived IL-25. FEBS J..

[28] Nonogaki K., Kaji T. (2020). Whey protein isolate inhibits hepatic FGF21 production, which precedes weight gain, hyperinsulinemia and hyperglycemia in mice fed a high-fat diet. Sci. Rep..

[29] Nonogaki K., Kaji T. (2023). Ingestion of whey protein and β-conglycinin exerts opposite effects on intestinal FGF15 and serotonin secretion in mice. Front. Endocrinol..

